# Mixed reality assisted target localization for transcranial magnetic stimulation navigation: a feasibility study

**DOI:** 10.1186/s12984-026-02045-z

**Published:** 2026-06-10

**Authors:** Zhongjie Shi, Zhengbo Yuan, Lu Gao, Yahui Hu, Guoxin Ni, Wenhui Liao, Yuting Xie, Jiawei He, Deyong Xiao, Xueqin Chen, Zhanxiang Wang

**Affiliations:** 1 Department of Neurosurgery, School of Medicine, The First Affiliated Hospital of Xiamen University, Xiamen University, Xiamen, 361003 China; 2 National Institute for Data Science in Health and Medicine, Xiamen University, Xiamen, 361003 China; 3 Department of Rehabilitation Medicine, School of Medicine, The First Affiliated Hospital of Xiamen University, Xiamen University, Xiamen, 361003 China; 4 Department of Scientific Research, School of Medicine, The First Affiliated Hospital of Xiamen University, Xiamen University, Xiamen, 361003 China

**Keywords:** Mixed reality (MR), Transcranial magnetic stimulation (TMS), Neuromodulation, Navigation

## Abstract

**Background:**

Transcranial magnetic stimulation (TMS), as a non-invasive neurostimulation technique, modulates neural activity by applying electromagnetic fields to specific areas of the brain. It is clinically used for several approved indications, including major depressive disorder, obsessive‑compulsive disorder, and migraine with aura, and is under active investigation for other neurological and psychiatric conditions. Accurate stimulation targeting is crucial for the effectiveness of TMS. Existing targeting methods, such as generic brain localization caps and the international 10–20 electroencephalogram (EEG) system, generally provide only rough localization, leading to significant targeting errors. In recent years, significant progress has been made in the application of mixed reality (MR) technology in medicine, particularly in surgical navigation, offering new ideas and possibilities for developing a simple, low-cost, and efficient TMS navigation system.

**Objective:**

This study proposes, for the first time, a portable MR navigation system for non-invasive neural modulation target localization. The aim is to evaluate its localization accuracy and operational efficiency in TMS through preclinical validation. This system seeks to provide a simple and high-precision localization solution for other non-invasive technologies, with the goal of improving localization accuracy and simplifying the operational workflow in clinical applications.

**Methods:**

The system is based on Microsoft HoloLens 2 and features three specifically designed interaction tools. Five different types of simulation head models were selected, and ten target points were set on each head model. CT scanning was used to obtain imaging data for each head model. Three researchers used the system to perform target localization and repeated the verification process by adjusting the head model posture (from standing to lying) to assess localization accuracy and efficiency.

**Results:**

The validation conducted by the three researchers showed the following results: In the standing position of the simulated head model, the measurement errors were 2.4 (IQR: 1.4–2.7) mm, 2.3 (IQR: 1.7–2.7) mm, and 2.6 (IQR: 1.9–3.0) mm, respectively. In the lying position of the simulated head model, the measurement errors were 1.9 (IQR: 1.6–2.4) mm, 2.0 (IQR: 1.4–3.0) mm, and 2.5 (IQR: 1.9–2.9) mm, respectively. There was a significant difference between researchers (*p* < 0.05), but no significant difference within the same researcher (*p* > 0.05).

**Conclusion:**

The TMS-Guide, based on mixed reality technology, is a portable and simple navigation solution that provides higher localization accuracy than traditional manual targeting. It shows promising potential for broader applications in non-invasive neural modulation and brain-computer interface fields.

## Introduction

In recent years, non‑invasive neuromodulation techniques have developed rapidly and are applied in various fields such as neural rehabilitation and the symptom alleviation of psychiatric disorders [1, 2]. These techniques include transcranial magnetic stimulation (TMS), transcranial electrical stimulation (TES), and transcranial ultrasound stimulation (TUS), which interact with the nervous system without the need for surgery. In contrast, invasive approaches—such as deep brain stimulation (DBS)—require surgically implanted electrodes and demand extremely high positioning precision, typically within the sub-millimeter range, to ensure effective stimulation and avoid damage to surrounding brain regions [3–5]. The main advantages of non-invasive technologies are lower risk, simpler operation, and greater flexibility.

As a representative neurostimulation technique, transcranial magnetic stimulation (TMS) uses a current-carrying coil to generate an alternating magnetic field that penetrates the skull and induces excitability changes in the cortical brain areas [6, 7]. TMS has important applications in neuroscience research and clinical practice. It is approved for the treatment of major depressive disorder, obsessive‑compulsive disorder, and migraine with aura, and has shown promise in modulating brain functions for a range of other neurological and psychiatric conditions (e.g., anxiety disorders, post‑stroke motor deficits), although many of these remain investigational [8, 9]. The clinical efficacy of TMS is influenced by multiple factors, among which precise target localization has been identified as a critical determinant of therapeutic outcomes [10]. However, common targeting methods, such as scalp localization based on the international 10–20 EEG system, have substantial spatial errors that affect targeting accuracy [11, 12].

With the continuous advancement of neuroimaging techniques, the application of structural magnetic resonance imaging (sMRI), functional magnetic resonance imaging (fMRI), and positron emission tomography (PET) has gradually improved the precision of TMS target localization. These imaging techniques can accurately identify brain regions associated with diseases, providing more reliable reference points for TMS [13]. Additionally, the advent of neuro-navigation has significantly improved TMS targeting accuracy. By combining fMRI of the patient with optical/electromagnetic tracking on the scalp, neuro-navigation achieves real-time spatial calibration of the coil, reducing localization errors to 1–2 mm [14]. Despite the significant improvement in TMS efficacy through neuro-navigation, its high cost, complex operation, and large equipment size still limit its adoption in primary care institutions [15]. Of course, integrated neuro-navigation systems have emerged, where the TMS device and the navigation system are presented as a single unit with a significantly reduced size. However, this may increase costs, so in some areas with limited medical resources, standalone TMS devices and localization caps are still more commonly used.

In recent years, mixed reality (MR)-based medical navigation systems have emerged as an innovative technology with potential [16, 17]. Unlike traditional targeting methods, MR technology provides a seamless, immersive navigation experience by combining virtual models with the real environment, enabling precise spatial mapping of complex brain structures through three-dimensional visualization that integrates virtual and real elements [18–20].

Although MR technology has been extensively studied and applied in surgical navigation with positive results, its application process in surgery remains complex due to strict sterile environment requirements and diverse patient postures [21]. Additionally, the long-duration use of MR glasses by surgeons during operations presents certain inconveniences, such as head and neck discomfort, dizziness [22]. In contrast to surgical navigation, MR has unique advantages when applied to non-invasive TMS targeting. TMS procedures do not require a sterile environment or head fixation, and the patient’s face remains unobstructed, allowing for simple sitting or lying postures. Moreover, the target for TMS is typically a functional brain region (e.g., the motor cortex or dorsolateral prefrontal cortex) rather than a single point. This inherent spatial tolerance—where stimulation efficacy is maintained over a cortical area—means that clinically useful accuracy does not demand sub-millimeter precision. However, conventional localization methods (e.g., the 10–20 EEG system) often yield substantial targeting errors that can exceed the acceptable range for optimal clinical outcomes [23]. MR-based navigation can bridge this gap by offering improved accuracy with a portable, intuitive, and workflow-friendly solution specifically tailored to TMS.

However, despite these advantages, to our knowledge, no reports have yet applied MR navigation to assist in the target localization of non-invasive neural modulation. Therefore, this study proposes and develops the first portable real-time auxiliary navigation system—TMS-Guide—based on MR technology. The goal is to fill this gap and demonstrate its potential to improve localization accuracy and simplify the operational process.

TMS-Guide is specifically designed for TMS target localization and demonstrates good scalability, suggesting its potential applicability to other non-invasive neuromodulation technologies. The system utilizes real-time head tracking and overlays three-dimensional anatomical models to assist in personalized TMS target localization. It simultaneously displays the patient’s head position and the virtual 3D anatomical model, dynamically adjusting the patient’s posture to ensure that TMS stimulation is accurately applied to the target brain area.

This study will focus on the design and implementation of the TMS-Guide system, evaluating its accuracy and overall performance through preclinical validation using simulation head models. The primary objective of this study is to provide a novel reference solution for non-invasive neural modulation target localization, offering a high-precision alternative to traditional manual methods or generic localization caps in the absence of neuro-navigation systems.

## Materials and methods

### System design

**Fig. 1:**
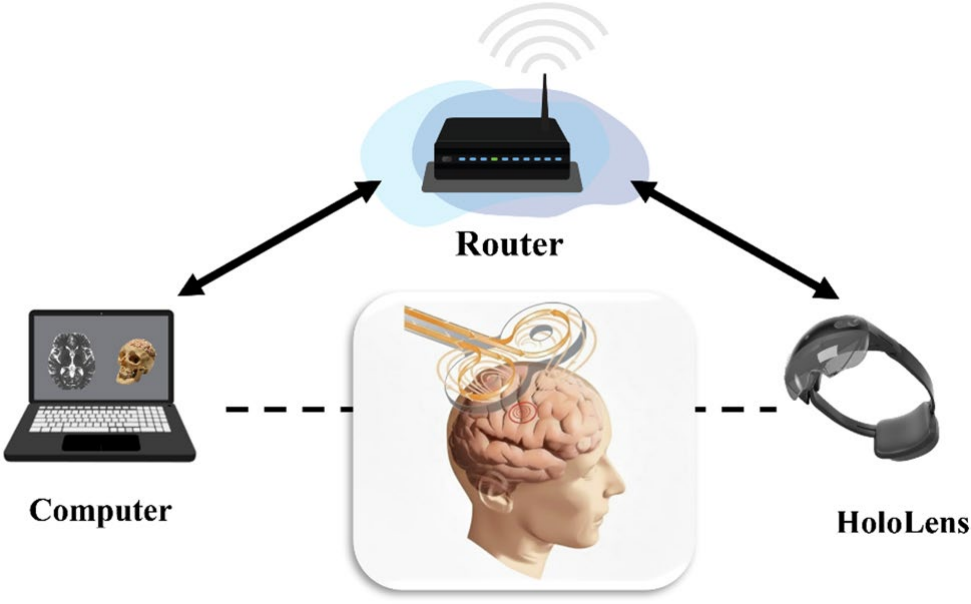
Structure of the TMS-Guide hardware and information transmission components

TMS-Guide system consists of both hardware and software components. Figure 1 illustrates the structure of the hardware and information transmission components. The computer, functioning as a mobile workstation, is responsible for processing medical imaging data and generating three-dimensional models. The HoloLens 2 (HL2), a commercial mixed-reality head-mounted display (HMD), integrates various sensors, such as Time of Flight (TOF) infrared depth sensors, eye-tracking cameras, and environmental cameras. It supports Wi-Fi and Bluetooth connectivity and utilizes a full-color waveguide display to provide real-depth perception of virtual objects. HL2 is used to display mixed-reality scenes and scan and recognize specific tools in the physical space. A local area network router establishes a network connection between the computer and HL2. During program operation, the computer transmits the three-dimensional models in real-time to HL2, while HL2 simultaneously transmits the first-person view back to the computer for display.

**Fig. 2:**
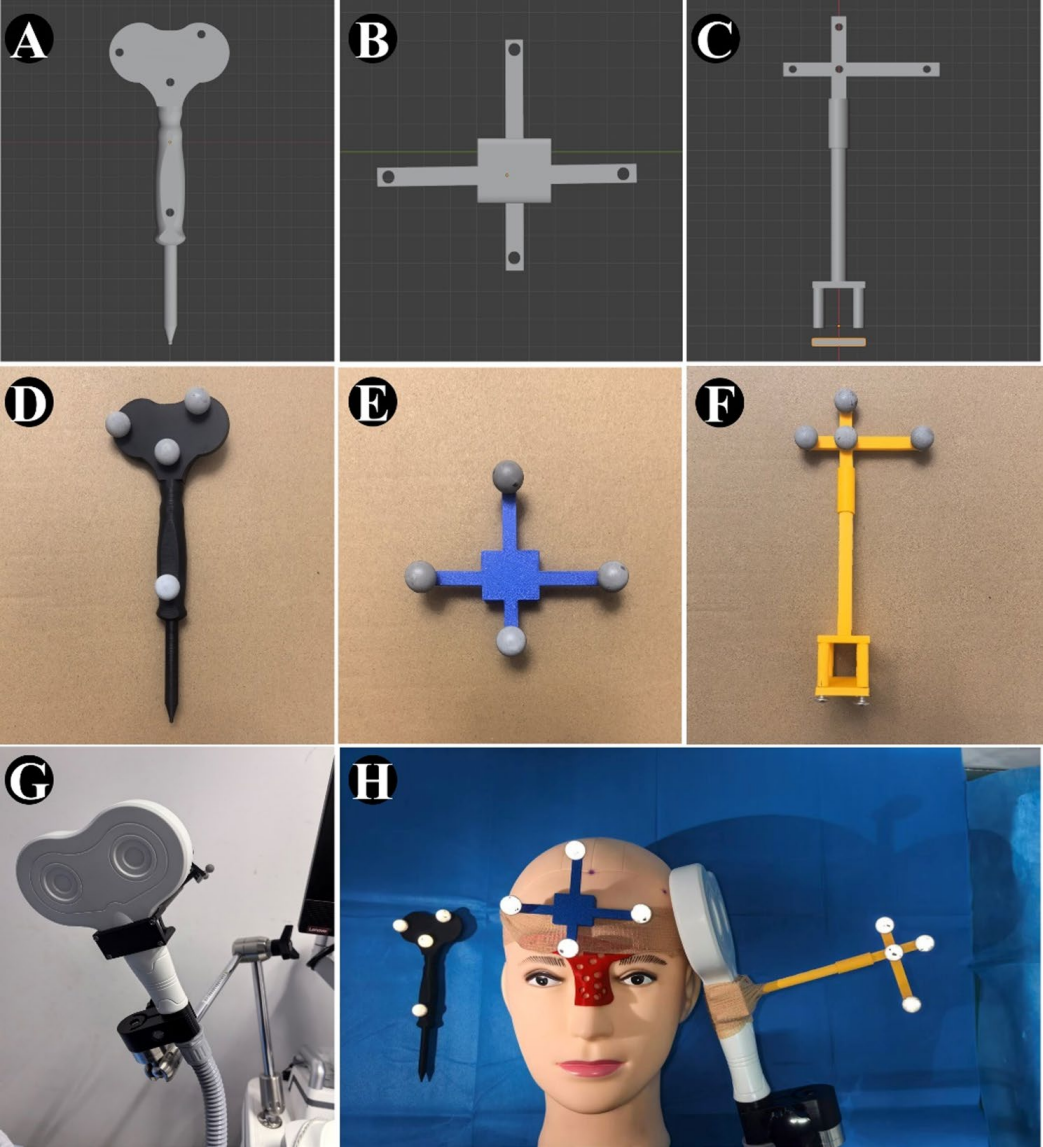
Auxiliary tools for the TMS device.** A**–**C** Design models of the three tools: registration tool, head tracking tool, and stimulator tool. **D-F** The three tools fabricated using 3D printing. **G** The TMS coil used in the study. **H** The usage of the three tools

To optimize the targeting process of the TMS device (YIRUIDE, Wuhan, China) used in this study, we designed three auxiliary tools: the Registration Tool, Head Tracking Tool, and Stimulator Tool. The Registration Tool is used to select feature points on the patient’s face for aligning the virtual model with the patient’s spatial coordinates. The Head Tracking Tool is bound to the patient to track the patient’s position in real-time. The Stimulator Tool is bound to the TMS coil to track the position of the coil in real-time. All of these tools were modeled and designed using Blender (5.0.0) software and 3D printed using polylactic acid (PLA) material. Each tool is equipped with four asymmetrical high-reflective balls, which are easily recognizable under near-infrared light and serve as markers for positioning and tracking (Fig. 2).

The TMS-Guide main program is developed based on Unity (version 2021.3.39f1), using Microsoft’s official MRTK tools (https://github.com/microsoft/MixedRealityToolkit-Unity). During system design, the original model parameters of the three tools are pre-stored within the software. Using the built-in cameras of HL2, the system can scan the position of tools in the physical space in real-time and perform localization based on the spatial coordinates of the high-reflective balls. By capturing the position of the high-reflective balls, the system matches the tools with the pre-stored models, allowing for the identification of specific tools. Once the tool is successfully identified, the virtual model is registered in real-time with the physical tool in the space, ensuring precise synchronization between the virtual and physical tool models.

### Data processing

**Fig. 3:**
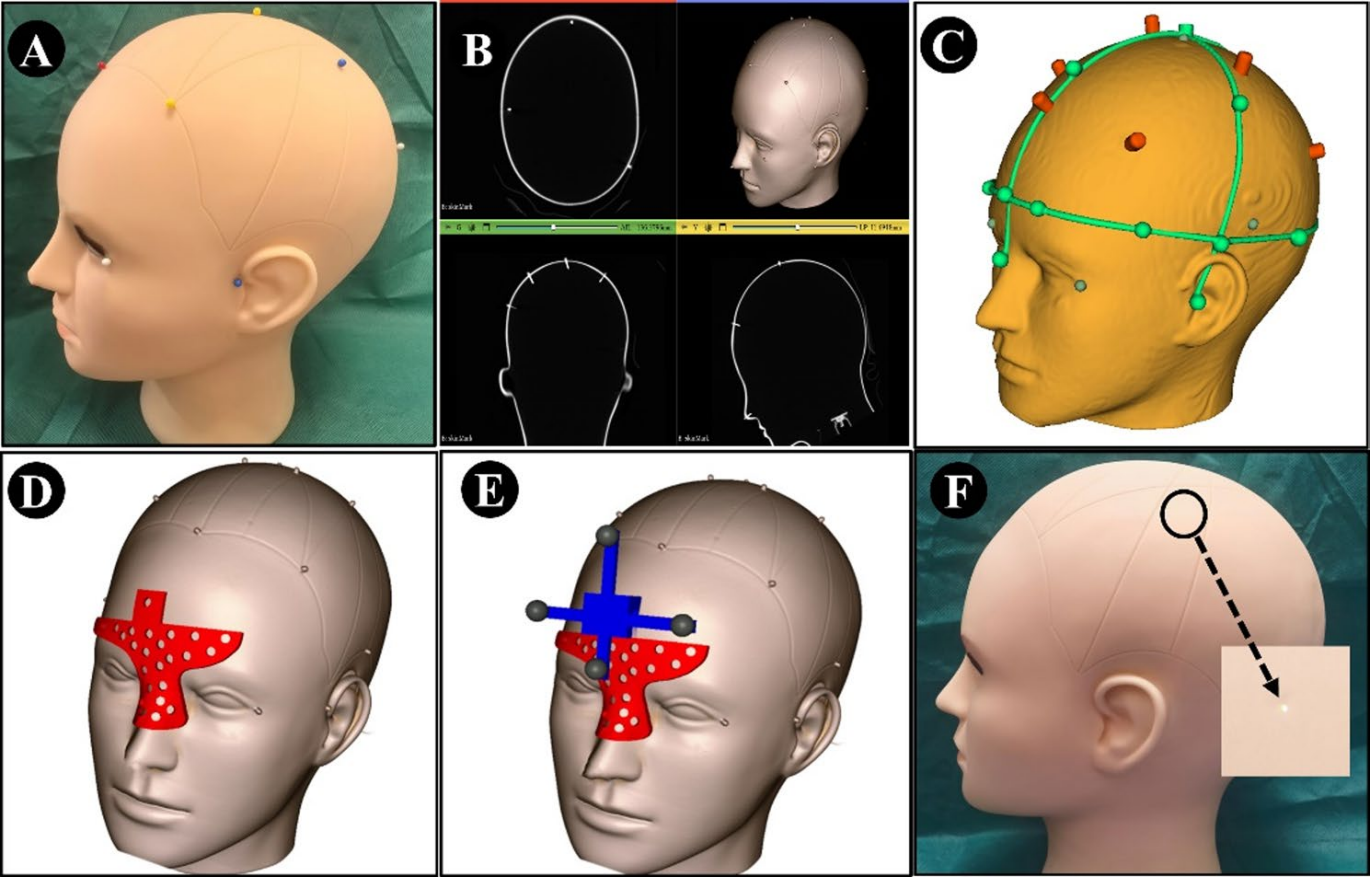
Data processing workflow and tool usage.** A**, **B** Spherical markers placed on the surface of the simulation head model, with DICOM data obtained through CT scanning. **C** Three-dimensional model reconstruction using 3D Slicer based on localization requirements. **D** After removing the spherical markers, tiny pinholes are retained on the model surface for identifying the marker positions.** E**,** F** A patient-specific facial template (personalized panel) was designed based on the reconstructed model. The template conforms to the facial surface and can be reproducibly positioned on the patient. It is rigidly attached to the Head Tracking Tool, enabling consistent spatial alignment between the tracking device and the patient across different sessions

To evaluate the localization accuracy of TMS-Guide, five different types of simulation head models were selected, and each model was randomly numbered. Ten spherical localization pins were uniformly fixed on the surface of each simulation head model, distributed across different areas and labeled using a numbering system. Subsequently, all models were scanned using a 64-row spiral CT scanner (Siemens SOMATOM, Germany) in high-resolution mode. The slice thickness was set to 0.625 mm, with continuous scanning slices covering the entire simulation head model to ensure accurate capture of each localization pin’s position. The scanning parameters were as follows: tube voltage of 120 kV, tube current of 200–300 mA, matrix size of 512 × 512 pixels, and the images were saved in DICOM format for subsequent processing and analysis (Fig. 3A-B).

An experienced TMS technician then imported each CT dataset into 3D Slicer software for three-dimensional reconstruction [24]. The reconstructed structure, including skin, marking lines, and spherical localization pins, was built using the threshold segmentation module (Fig. 3C). To facilitate spatial alignment for the patient throughout the therapy cycle, a personalized panel was designed for each simulation head model. This panel was uniquely matched and fixed with the Head Tracking Tool. The main purpose of this design was to achieve spatial alignment for the patient during the first treatment session, and for subsequent treatments, the personalized panel could be worn to automatically align the model in space (Fig. 3D-E). Finally, the reconstructed skin model and marking point model were exported in OBJ format, preparing them for the subsequent MR navigation application. To avoid interference from the tiny pinholes of the spherical localization pins on the simulation head model surface during MR navigation, these pins were removed. Although the pinholes were visible to the naked eye, they were not directly observable during MR navigation, ensuring that they would not affect the localization process (Fig. 3F).

**Fig. 4:**
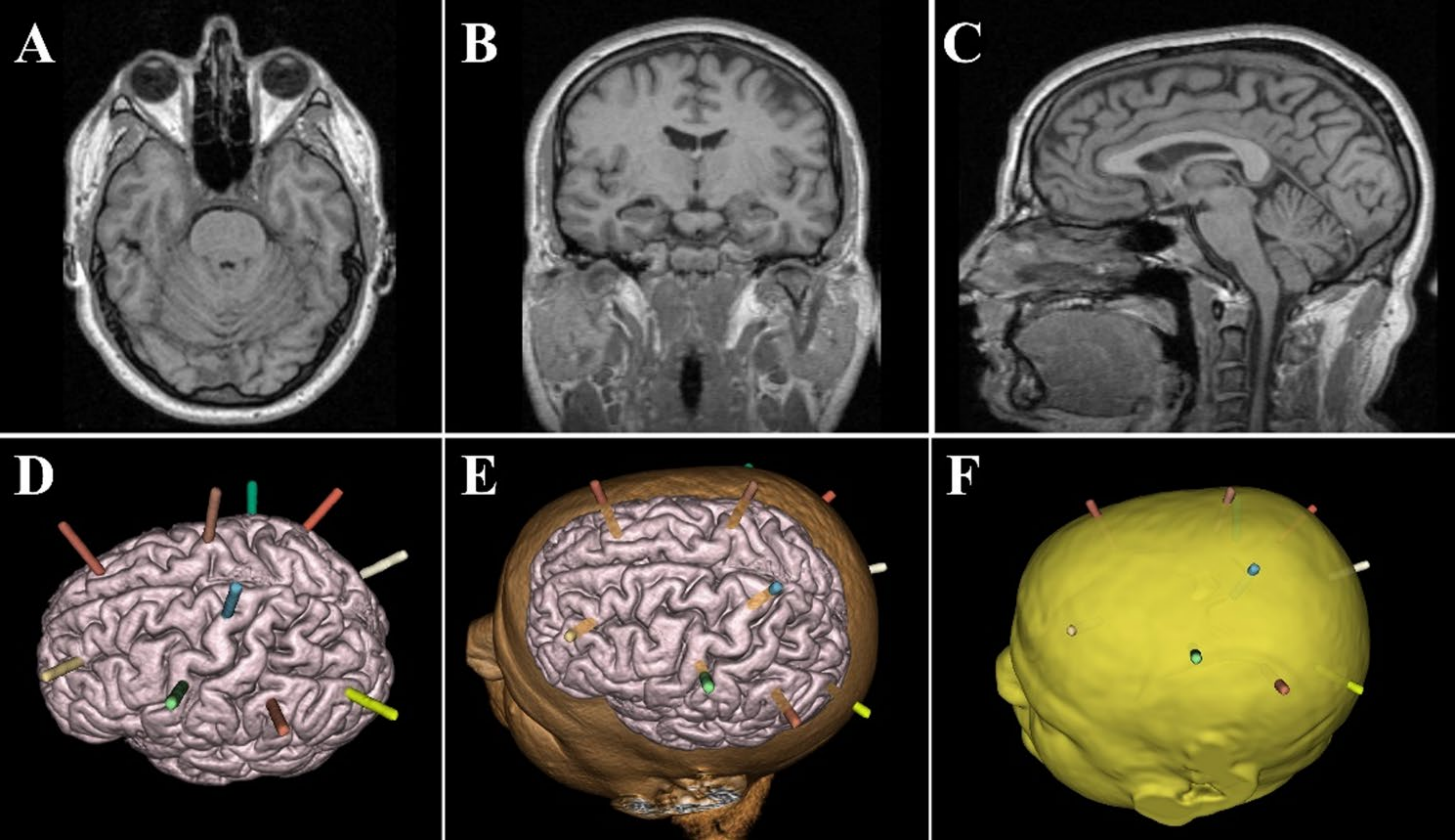
MRI-based brain segmentation and 3D reconstruction.** A**–**C** Cross-sectional, coronal, and sagittal views of cranial MRI.** D**–**F** MRI-based brain segmentation, TMS multi-target point marking, and 3D model creation

It should be noted that in clinical applications, if the patient’s original imaging data is MRI, the data processing workflow will be the same as that of CT imaging. However, MRI data provides more accurate brain region segmentation, particularly in the delineation of sulci and gyri. This advantage enables more precise target area setting and allows for multi-target precise localization based on the specific locations of the sulci and gyri (Fig. 4). By using this method, patients only need to complete modeling for all target settings once during the entire treatment cycle, avoiding the need for repeated modeling due to target adjustments during treatment. This not only saves time but also improves treatment efficiency, reduces repetitive operations, and optimizes the clinical treatment process.

### Preclinical evaluation

Three researchers (A, B, C) randomly selected a simulation head model and imported its corresponding 3D model into the TMS-Guide system. The Head Tracking Tool was then fixed onto the simulation head model using medical elastic bandages. Next, the Registration Tool was used to select four facial landmarks on the patient’s face—such as the nasal tip, left canthus, right canthus, and tragus—to perform point-based registration between the virtual model and the simulated head model. Notably, in practical applications, the selected registration points should be spatially well distributed and should avoid being coplanar as much as possible. This point-based registration method has been validated in previous mixed reality navigation studies and this study specifically evaluated the changes in localization errors and repeatability before and after head position changes [25, 26].

During the experiment, the position of 10 spherical localization pins was sequentially located. Each localization point was marked with an erasable marker, and the Euclidean distance between each marked point and the real localization pin was measured and considered as the localization error. Each point was measured three times, and the mean value was taken as the final error. The posture of the simulation head model was then adjusted from standing to lying position, and localization was repeated to evaluate the effect of head position changes on localization accuracy. The entire process was timed until all researchers completed the validation for five head models. All researchers received a 30-minute training session before the experiment and restarted the system before each localization to ensure the software was completely reset. Data measurement and recording were performed by independent researchers.

### Statistical analysis

All statistical analyses were conducted using Python 3.12. The normality of the data was first assessed using the Shapiro-Wilk test. Quantitative data that followed a normal distribution were expressed as mean ± standard deviation (SD); otherwise, the median and interquartile range (IQR) were reported. The time taken for each researcher to complete the localization was primarily analyzed using descriptive statistics. Differences within groups were assessed using paired t-tests, and, for non-normally distributed data, the Wilcoxon signed-rank test was applied. Between-group differences in localization errors were compared using repeated measures ANOVA or Friedman’s test, depending on the distribution of the data. A significance level of *p* < 0.05 (two-tailed) was considered statistically significant for all tests. All statistical analyses were exploratory, consistent with the conceptual validation design of this study.

## Results

**Fig. 5:**
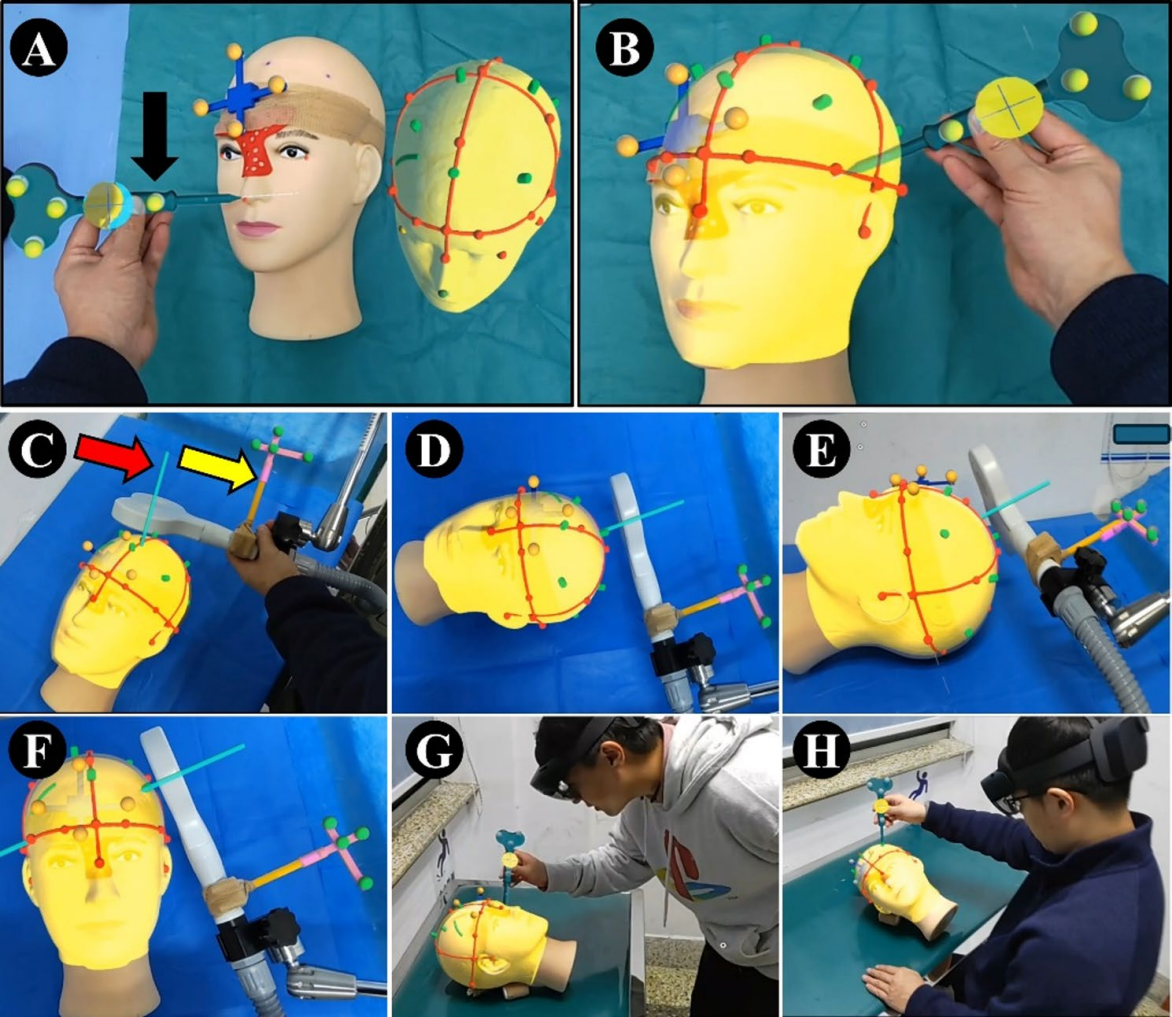
TMS-Guide localization process and tool usage examples.** A**,** B** Registration process from the virtual model to the patient. In panel** A**, the black arrow indicates the researcher holding the Registration Tool to select feature points on the patient’s face. Panel** B** shows the result after registration.** C**–**F** Real-time tracking of the TMS coil and localization after patient head position change. In panel** C**, both the red and yellow arrows indicate the components of the Stimulator Tool. The red arrow points to the virtual probe model located at the center of the TMS coil, which is used to guide the optimal direction of the current TMS coil.** G**,** H** Mixed reality scene from a third-person perspective, with the researcher observing through HL2

During usage, TMS-Guide was able to stably track the three localization tools simultaneously. Figure 5 illustrates the complete target localization process.

The three-dimensional models were created using 3D Slicer software, with each model taking approximately 10 min to complete. Three researchers performed localization on five head models, totaling 50 target points. The time taken for each researcher to complete the localization was as follows: Researcher A: 7.0 ± 0.9 min (range: 5.9–8.1 min); Researcher B: 6.8 ± 1.1 min (range: 5.4–7.8 min); Researcher C: 6.2 ± 1.3 min (range: 5.2–8.5 min).

Three researchers performed measurements on the simulation head models in both standing and lying postures. The Shapiro-Wilk test showed that all measurement results were not normally distributed (*p* < 0.05), so the Wilcoxon signed-rank test was used for within-group comparisons. The results indicated that there were no significant differences in the measurements among the three researchers in both standing and lying postures (*p* > 0.05). Table 1 summarizes the measurement results of the three researchers in different postures.

**Table 1:** Measurement results of three researchers

Researcher	Model pose	Error (mm)	Range (mm)	95%CI (mm)	*P*-value
A	Standing position	2.4(IQR: 1.4.-2.7)	0.9–3.6	1.9–2.3	0.31
	Supine position	1.9(IQR: 1.6.-2.4)	1.1–3.3	1.8–2.1	
B	Standing position	2.3(IQR: 1.7.-2.7)	1.2–3.1	2.0-2.4	0.53
	Supine position	2.0(IQR: 1.4.-3.0)	0.7–3.4	1.9–2.3	
C	Standing position	2.6(IQR: 1.9.-3.0)	1.2–3.5	2.3–2.7	0.15
	Supine position	2.5(IQR: 1.9.-2.9)	1.3–3.3	2.3–2.6	

**Fig. 6:**
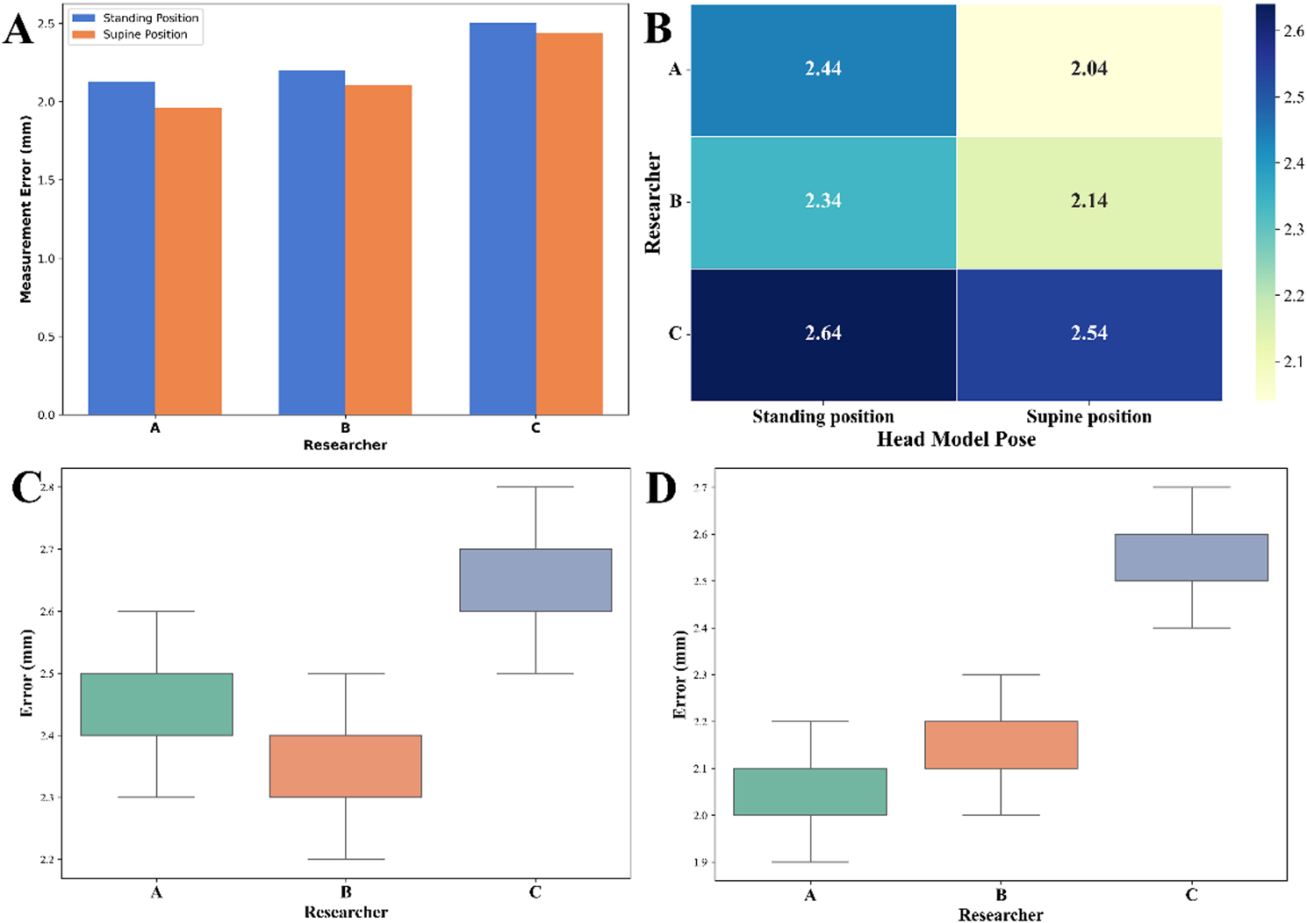
Comparison of researcher measurement results. **A** Bar chart of measurement results from the three researchers. **B** Heatmap of measurements by the three researchers under different conditions.** C**,** D** Box plots of measurement results from the three researchers in standing and lying postures of the simulation head model

Further analysis was conducted using the Friedman test to compare the measurements between the three researchers. The results showed that, in both the standing and lying postures of the simulation head model, there were significant differences in the measurement results between the researchers (*p* < 0.001) (Fig. 6). Specifically, the localization errors of Researcher C were consistently higher than those of the other two researchers in both postures.

## Discussion

This study aims to design and evaluate a MR-based non-invasive neural modulation target localization solution. We developed a simple TMS-Guide navigation system, specifically optimized for the TMS application process. Preclinical evaluation is essential before formal clinical assessment. Using simulation head models, we validated the system’s performance in localization accuracy and efficiency. The results preliminarily demonstrate that researchers are able to achieve millimeter-level precise localization of multiple target points in different postures in a short amount of time and the system operates stably, exhibiting high engineering reliability.

TMS-Guide is primarily designed to assist doctors in localizing the target area for TMS, rather than identifying the most appropriate treatment target for the individual patient. The process of determining an individualized, optimal target area often requires complementary technologies such as fMRI, NIRS, and brain network analysis. However, TMS-Guide is focused on accurately mapping the target region, which has been identified through these analyses, onto the patient’s actual head position. This enables the precise placement of the TMS coil, ensuring better guidance for the doctor during the procedure.

The TMS-Guide presented in this paper is a novel navigation technology that offers advantages including portability, ease of use (with new users quickly adapting and minimal localization time), and low cost. Although existing neural navigation systems are considered the gold standard for improving TMS coil target localization accuracy, numerous practical operational barriers may hinder the application of this technology in many treatment environments [23]. This is especially true outside academic or research centers, where the large size of standalone neural navigation equipment makes it difficult to move and use in different locations. Methods such as generic localization caps, the international 10–20 EEG system, and Beam F3 can be used to locate specific common targets. In the absence of neural navigation, these methods can offer some assistance and do not require additional patient imaging scans [27]. However, these methods rely on the operator’s skill, and their repeatability may vary depending on the operator and repeated measurements. Moreover, these methods lack individualized design, which may reduce the effectiveness of TMS treatment [28, 29].

Earlier and recent studies have reported on personalized TMS target localization solutions based on 3D-printed helmets, achieving good localization accuracy [30, 31]. However, since 3D printing technology uses a unidirectional design, once the helmet is printed, adjustments to the target area during use require redesigning and printing a new helmet. Additionally, the printing process is time-consuming, and there is a certain failure rate in printing [32]. Therefore, compared to mixed reality navigation technology, 3D printing may lack flexibility. Furthermore, MR navigation is not limited by the complexity of the model when displaying three-dimensional models, and it can simultaneously show important structures such as sulci and gyri. In contrast, 3D printing only displays the projection of the target area on the scalp, lacking the immersive experience and rich visual effects [33].

In this study, the time spent by all researchers on localization did not exceed 10 min. It is important to note that the study time included localization in both standing and lying postures of the simulation head model, with 10 target points set for each posture, and each point was measured three times. Therefore, in practical applications, the localization time may be shorter, highlighting the efficiency of the TMS-Guide system. In terms of localization accuracy, there were no significant differences in the measurement errors of the three researchers in both the standing and lying postures, which partially indicates that the Head Tracking Tool can achieve stable tracking of the patient’s head even with movement, without being affected by the extent of the movement. Additionally, in actual clinical use, the patient’s head position is generally relatively fixed, with only minimal large-scale changes from standing to lying posture. Despite the unified training provided to all researchers, significant differences between researchers were still observed in the actual operation, though these differences did not exceed 1 mm, demonstrating good repeatability. This suggests that the TMS-Guide system is unlikely to result in operator errors between different users, likely due to its standardized operating procedure and real-time visual reference information. While training helps reduce operational differences, individual factors such as operating habits, hand-eye coordination, and experience may still have an impact on measurement results [34]. Furthermore, as a portable navigation system running on HL2, TMS-Guide relies on HL2, a device powered by lithium batteries, and the depletion of the battery power can lead to a gradual decrease in system performance [35]. Consequently, when operating under low-battery conditions, the system’s localization error may increase.

The main limitation of this study is that it is a preclinical validation with a small sample size. Therefore, larger-scale, multi-center clinical patient evaluations are still needed before formal clinical application, and this work is beyond the scope of this study. In addition, this study was conducted using a head phantom and CT data acquired from the phantom. Future studies involving healthy volunteers or patients, as well as MRI-based anatomical data, are therefore warranted to further assess the clinical feasibility and generalizability of the proposed system. Another limitation is that different TMS systems use different types of coils. The Stimulator Tool designed in this study is tailored to a specific coil. Nevertheless, extending the system to other coil types is feasible and straightforward. Prolonged use of head-mounted devices may cause discomfort to the operator. Although this was not observed in the present study, it is important to explore the development of more portable and lightweight headsets. Another limitation of this study is that the current hardware platform is relatively homogeneous, being confined to the HoloLens 2. However, we have already begun evaluating new devices, specifically the SeerLens series developed by Xvisio Technology (Shanghai, China). In future iterations of the system, we plan to conduct additional tests using these emerging MR devices. In addition to the aforementioned limitation, the study design did not take into account the impact of different target point selections on localization accuracy.

Future research should expand the sample size and conduct multi-center clinical studies to validate the effectiveness and safety of the system in a broader context. Additionally, the current system needs optimization, especially in handling patient head position changes. A feature could be developed where, when the TMS coil position deviates from the target area beyond a certain angle, the system automatically sends an alert to remind the operator or patient to adjust the position. Based on the prototype design in this study, future work could consider developing a more user-friendly version that features an adjustable Stimulator Tool to cater to different clinical needs. For the alignment of virtual models with real patients, future efforts could focus on developing an automatic alignment solution based on facial recognition, reducing human error and improving localization accuracy.

## Conclusion

The mixed reality navigation system developed for TMS demonstrated simplicity, reliability, and accuracy. With further optimization and refinement, the system may be extended to other neuromodulation procedures, including transcranial electrical stimulation (TES), transcranial ultrasound stimulation (TUS), among others. Although the current TMS-Guide described in this study is still in the prototype stage, improving it for routine and convenient use in clinical populations is not a complex task.

## Data Availability

The complete software and full tool model files will be shared upon reasonable request for the purpose of replication by contacting the corresponding author.

## References

[1] Albantakis L, Tononi G. Precision neuromodulation in psychiatry: focus on temporal interference stimulation. Am J Psychiatry. 2026;appiajp20250873. Advance online publication. 10.1176/appi.ajp.20250873. 10.1176/appi.ajp.20250873 41922984

[2] Hu Q, Jiao X, Ding Y, Wei Y, Tang X, Xu L, Cui H, Tang Y, Liu H, Gao J, Zeng L, Yi Z, Li C, Chen J, Wang J, Zhang T. The effects of rTMS over orbitofrontal cortex on cognitive functions in first-episode schizophrenia. Psychol Med. 2026;56:e119. 10.1017/S0033291726103912. 42046881 10.1017/S0033291726103912 PMC13125931

[3] Hariz M, Blomstedt P. Deep brain stimulation for Parkinson’s disease. J Intern Med. 2022;292(5):764–78. 10.1111/joim.13541. 35798568 10.1111/joim.13541 PMC9796446

[4] David R, Scala MR, Ellenbogen J. Review of the targeting accuracy of frameless and frame-based robot-assisted deep brain stimulation electrode implantation in pediatric patients using the Neurolocate module. J Neurosurg Pediatr. 2023;33(3):207–13. 10.3171/2023.10.PEDS23275. 38134418 10.3171/2023.10.PEDS23275

[5] Rassoulou F, Sharma A, Steina A, Butz M, Hartmann CJ, Bahners BH, Vesper J, Schnitzler A, Hirschmann J. Electrophysiological signatures predict the therapeutic window of deep brain stimulation electrode contacts. NPJ Digit Med. 2025;8(1):635. 10.1038/s41746-025-02089-w. 41162751 10.1038/s41746-025-02089-w PMC12572235

[6] Klomjai W, Katz R, Lackmy-Vallée A. Basic principles of transcranial magnetic stimulation (TMS) and repetitive TMS (rTMS). Annals Phys rehabilitation Med. 2015;58(4):208–13. 10.1016/j.rehab.2015.05.005. 10.1016/j.rehab.2015.05.005 26319963

[7] Wang B, Wang Y, Yang F, Han F, Li K, Sun K, Liang P. The effect of repetitive transcranial magnetic stimulation on immediate and long-term cognitive functions in Alzheimer’s dementia and mild cognitive impairment: a meta-analysis. J Neuroeng Rehabil. 2025;22(1):262. 10.1186/s12984-025-01777-8. 41398677 10.1186/s12984-025-01777-8 PMC12707008

[8] Cappon D, den Boer T, Jordan C, Yu W, Metzger E, Pascual-Leone A. Transcranial magnetic stimulation (TMS) for geriatric depression. Ageing Res Rev. 2022;74:101531. 10.1016/j.arr.2021.101531. 34839043 10.1016/j.arr.2021.101531 PMC8996329

[9] Koutsomitros T, Evagorou O, Schuhmann T, Zamar A, Sack AT. Advances in transcranial magnetic stimulation (TMS) and its applications in resistant depression. Psychiatriki. 2021;32(Supplement I):90–8. 10.22365/jpsych.2021.054. 34990384 10.22365/jpsych.2021.054

[10] Liu B, Hu C, Bao P. Precision TMS through the integration of neuroimaging and machine learning: optimizing stimulation targets for personalized treatment. Front Hum Neurosci. 2025;19:1682852. 10.3389/fnhum.2025.1682852. 41089381 10.3389/fnhum.2025.1682852 PMC12515893

[11] Wang Y, Gotman J. The influence of electrode location errors on EEG dipole source localization with a realistic head model. Clin neurophysiology: official J Int Federation Clin Neurophysiol. 2001;112(9):1777–80. 10.1016/s1388-2457(01)00594-6. 10.1016/s1388-2457(01)00594-6 11514261

[12] Scrivener CL, Reader AT. Variability of EEG electrode positions and their underlying brain regions: visualizing gel artifacts from a simultaneous EEG-fMRI dataset. Brain Behav. 2022;12(2):e2476. 10.1002/brb3.2476. 35040596 10.1002/brb3.2476 PMC8865144

[13] Duprat RJ, Linn KA, Satterthwaite TD, Sheline YI, Liang X, Bagdon G, Flounders MW, Robinson H, Platt M, Kable J, Long H, Scully M, Deluisi JA, Thase M, Cristancho M, Grier J, Blaine C, Figueroa-González A, Oathes DJ. Resting fMRI-guided TMS evokes subgenual anterior cingulate response in depression. NeuroImage. 2025;305:120963. 10.1016/j.neuroimage.2024.120963. 39638081 10.1016/j.neuroimage.2024.120963 PMC11887861

[14] Demchenko I, Al-Shamali HF, Rueda A, Tailor I, Janssen-Aguilar R, Schweizer TA, Dunlop K, Graham SJ, Bhat V. Magnetic Resonance Imaging-Guided Neuronavigation for Transcranial Magnetic Stimulation in Mood Disorders: Technical Foundation, Advances, and Emerging Tools. Hum Brain Mapp. 2025;46(17):e70424. 10.1002/hbm.70424. 41310980 10.1002/hbm.70424 PMC12660162

[15] Yu GH, Park C, Jeong MG, Jung GS, Kim KT. Clinical implementation, barriers, and unmet needs of rTMS and neuro-navigation systems in stroke rehabilitation: a nationwide survey in South Korea. Front Neurol. 2024;15:1423013. 10.3389/fneur.2024.1423013. 39139770 10.3389/fneur.2024.1423013 PMC11321079

[16] Cai Z, Zhou Y, Wen Z, Li L, Yi Y. A bibliometric analysis of augmented reality and mixed reality applied in minimally invasive surgery. J robotic Surg. 2025;19(1):159. 10.1007/s11701-025-02312-6. 10.1007/s11701-025-02312-6 40232554

[17] Yeung AWK, Tosevska A, Klager E, Eibensteiner F, Laxar D, Stoyanov J, Glisic M, Zeiner S, Kulnik ST, Crutzen R, Kimberger O, Kletecka-Pulker M, Atanasov AG, Willschke H. Virtual and Augmented Reality Applications in Medicine: Analysis of the Scientific Literature. J Med Internet Res. 2021;23(2):e25499. 10.2196/25499. 33565986 10.2196/25499 PMC7904394

[18] Lin CJ, Lin KC, Lau HY, Hsieh YW, Li YC, Chen WS, Chen CL, Chang YJ, Lee YY, Yao G, Hrong YS, Pan HC, Wu YH, Hsu WL, Kuo CC, Tsai HT, Lin CY, Chang PC. Effects of mirror therapy preceding augmented reality in stroke rehabilitation: a randomized controlled trial. J Neuroeng Rehabil. 2025. 10.1186/s12984-025-01820-8. Advance online publication. 41444616 10.1186/s12984-025-01820-8 PMC12961786

[19] Cho J, Rahimpour S, Cutler A, Goodwin CR, Lad SP, Codd P. Enhancing Reality: A Systematic Review of Augmented Reality in Neuronavigation and Education. World Neurosurg. 2020;139:186–95. 10.1016/j.wneu.2020.04.043. 32311561 10.1016/j.wneu.2020.04.043

[20] Bronowicki K, Antoniuk-Majchrzak J, Malesza I, Możarowski W, Szymborska A, Pachuta B, Walenta T, Jasica W, Stanuch M, Skalski A, Raciborska A. An attempt to evaluate the use of mixed reality in surgically treated pediatric oncology patients. NPJ Digit Med. 2025;8(1):262. 10.1038/s41746-025-01638-7. 40346298 10.1038/s41746-025-01638-7 PMC12064705

[21] Vervoorn MT, Wulfse M, Van Doormaal TPC, Ruurda JP, Van der Kaaij NP, De Heer LM. Mixed Reality in Modern Surgical and Interventional Practice: Narrative Review of the Literature. JMIR Serious Games. 2023;11:e41297. 10.2196/41297. Published 2023 Jan 6. 36607711 10.2196/41297 PMC9947976

[22] Magalhães R, Oliveira A, Terroso D, Vilaça A, Veloso R, Marques A, Pereira J, Coelho L. Mixed Reality in the Operating Room: A Systematic Review. J Med Syst. 2024;48(1):76. 10.1007/s10916-024-02095-7. 39145896 10.1007/s10916-024-02095-7 PMC11327191

[23] Caulfield KA, Fleischmann HH, Cox CE, Wolf JP, George MS, McTeague LM. Neuronavigation maximizes accuracy and precision in TMS positioning: Evidence from 11,230 distance, angle, and electric field modeling measurements. Brain Stimul. 2022;15(5):1192–205. 10.1016/j.brs.2022.08.013. 36031059 10.1016/j.brs.2022.08.013 PMC10026380

[24] Fedorov A, Beichel R, Kalpathy-Cramer J, Finet J, Fillion-Robin JC, Pujol S, Bauer C, Jennings D, Fennessy F, Sonka M, Buatti J, Aylward S, Miller JV, Pieper S, Kikinis R. 3D Slicer as an image computing platform for the Quantitative Imaging Network. Magn Reson Imaging. 2012;30(9):1323–41. 10.1016/j.mri.2012.05.001. 22770690 10.1016/j.mri.2012.05.001 PMC3466397

[25] Liu Z, Yang Z, Jiang S, Zhou Z. A Spatial Registration Method Based on Point Cloud and Deep Learning for Augmented Reality Neurosurgical Navigation. Int J Med Rob + Comput Assist surgery: MRCAS. 2024;20(6):e70030. 10.1002/rcs.70030. 10.1002/rcs.70030 39716403

[26] Morley CT, Arreola DM, Qian L, Lynn AL, Veigulis ZP, Osborne TF. Mixed Reality Surgical Navigation System; Positional Accuracy Based on Food and Drug Administration Standard. Surg Innov. 2024;31(1):48–57. 10.1177/15533506231217620. 38019844 10.1177/15533506231217620 PMC10773158

[27] Fabregat-Sanjuan A, Pàmies-Vilà R, Pascual-Rubio V. Evaluation of the Beam-F3 method for locating the F3 position from the 10–20 international system. Brain Stimul. 2022;15(4):1011–2. 10.1016/j.brs.2022.07.002. 35863653 10.1016/j.brs.2022.07.002

[28] Gomez LJ, Dannhauer M, Peterchev AV. Fast computational optimization of TMS coil placement for individualized electric field targeting. NeuroImage. 2021;228:117696. 10.1016/j.neuroimage.2020.117696. 33385544 10.1016/j.neuroimage.2020.117696 PMC7956218

[29] Rajasekharan D, Madore MR, Holtzheimer P, Lim KO, Williams LM, Philip NS. Personalized models of Beam/F3 targeting in transcranial magnetic stimulation for depression: Implications for precision clinical translation. Brain Stimul. 2025;18(3):829–37. 10.1016/j.brs.2025.04.003. 40194594 10.1016/j.brs.2025.04.003 PMC12119053

[30] Mansouri F, Mir-Moghtadaei A, Niranjan V, Wu JS, Akhmedjanov D, Nuh M, Cairo T, Giacobbe P, Zariffa J, Downar J. Development and validation of a 3D-printed neuronavigation headset for therapeutic brain stimulation. J Neural Eng. 2018;15(4):046034. 10.1088/1741-2552/aacb96. 29888708 10.1088/1741-2552/aacb96

[31] Wang H, Cui D, Jin J, Wang X, Li Y, Liu Z, Yin T. 3D-printed helmet-type neuro-navigation approach (I-Helmet) for transcranial magnetic stimulation. Front NeuroSci. 2023;17:1224800. 10.3389/fnins.2023.1224800. 37609452 10.3389/fnins.2023.1224800 PMC10442160

[32] Lu A, Williams RO 3rd, Maniruzzaman M. 3D printing of biologics-what has been accomplished to date? Drug Discov Today. 2024;29(1):103823. 10.1016/j.drudis.2023.103823. 10.1016/j.drudis.2023.103823 37949427

[33] Sandralegar A, Bernard F, Khatchatourov S, Janssen I, Schaller K, Bijlenga P, Haemmerli J. Mixed reality compared to the traditional ex cathedra format for neuroanatomy learning: the value of a three-dimensional virtual environment to better understand the real world. NeuroSurg Focus. 2024;56(1):E14. 10.3171/2023.10.FOCUS23620. 38163348 10.3171/2023.10.FOCUS23620

[34] Ríder-Vázquez A, Gutiérrez-Sánchez E, Martinez-Perez C, Sánchez-González MC. Test-Retest Reliability of a Computerized Hand-Eye Coordination Task. J eye Mov Res. 2025;18(5):54. 10.3390/jemr18050054. 41149956 10.3390/jemr18050054 PMC12565402

[35] Sahu D, Nidhi, Prakash S, Pandey VK, Yang T, Rathore RS, Wang L. Edge assisted energy optimization for mobile AR applications for enhanced battery life and performance. Sci Rep. 2025;15(1):10034. 10.1038/s41598-025-93731-w. 40122909 10.1038/s41598-025-93731-w PMC11930950

